# Influence of Anthropogenic Disturbances on Stand Structural Complexity in Andean Temperate Forests: Implications for Managing Key Habitat for Biodiversity

**DOI:** 10.1371/journal.pone.0169450

**Published:** 2017-01-09

**Authors:** Julián Caviedes, José Tomás Ibarra

**Affiliations:** 1 Centre for Local Development, Education and Interculturality (CEDEL), Villarrica Campus, Pontificia Universidad Católica de Chile, Villarrica, La Araucanía Region, Chile; 2 Fauna Australis Wildlife Laboratory, Department of Ecosystems and Environment, School of Agriculture and Forest Sciences, Pontificia Universidad Católica de Chile, Santiago, Chile; Technical University in Zvolen, SLOVAKIA

## Abstract

Forest attributes and their abundances define the stand structural complexity available as habitat for faunal biodiversity; however, intensive anthropogenic disturbances have the potential to degrade and simplify forest stands. In this paper we develop an index of stand structural complexity and show how anthropogenic disturbances, namely fire, logging, livestock, and their combined presence, affect stand structural complexity in a southern Global Biodiversity Hotspot. From 2011 to 2013, we measured forest structural attributes as well as the presence of anthropogenic disturbances in 505 plots in the Andean zone of the La Araucanía Region, Chile. In each plot, understory density, coarse woody debris, number of snags, tree diameter at breast height, and litter depth were measured, along with signs of the presence of anthropogenic disturbances. Ninety-five percent of the plots showed signs of anthropogenic disturbance (N = 475), with the combined presence of fire, logging, and livestock being the most common disturbance (N = 222; 44% of plots). The lowest values for the index were measured in plots combining fire, logging, and livestock. Undisturbed plots and plots with the presence of relatively old fires (> 70 years) showed the highest values for the index of stand structural complexity. Our results suggest that secondary forests < 70-year post-fire event, with the presence of habitat legacies (e.g. snags and CWD), can reach a structural complexity as high as undisturbed plots. Temperate forests should be managed to retain structural attributes, including understory density (7.2 ± 2.5 # contacts), volume of CWD (22.4 ± 25.8 m^3^/ha), snag density (94.4 ± 71.0 stems/ha), stand basal area (61.2 ± 31.4 m^2^/ha), and litter depth (7.5 ± 2.7 cm). Achieving these values will increase forest structural complexity, likely benefiting a range of faunal species in South American temperate forests.

## Introduction

Forests are complex systems composed by multiple attributes that interact with each other across different spatial levels [1]. At the stand-level, structural complexity is the measure of a number of attributes present in a forest stand and the relative abundance of each of these attributes [2]. Studies have shown that stands with higher structural complexity generally harbor higher species diversity while promoting greater ecosystem functioning and stability in comparison to less complex ones [3–6]. Stand structural complexity can vary among different habitat types and successional stages [4,7,8]. For example, old-growth forests are positively related with stand structural complexity in Douglas-fir forests in North America [7]. However, structural complexity in certain secondary forest stages may be higher than in old-growth stands due to the presence of structural habitat legacies (i.e. standing dead trees, or snags, and coarse woody debris) remaining after disturbance [8,9].

Modification and adaptation of forests after disturbances make the quantification of structural attributes an extremely complicated task. Therefore, the combination of different key structural attributes into an index of stand structural complexity is a practical approach to guide forest conservation and management [2,3,10,11]. The rationale behind using an index of stand structural complexity is that higher values for the index indicate a more complex array (e.g. higher presence and relative abundances) of structural attributes, providing greater niches for a broad array of organisms while ameliorating negative relationships among them, such as depredation and competition [12–15].

Intensive anthropogenic disturbances, including fire, logging, and livestock farming have the potential to degrade the composition and availability of structural attributes in forests [16–18]. These disturbances can degrade the density of the understory, volume of coarse woody debris (CWD), density of snags, stand basal area, and litter depth [16–20]. The loss of these forest attributes are known to affect biodiversity including mammals, birds, reptiles, amphibians, and invertebrates that depend on these key structural habitat attributes for their survival [21–24]. For instance, a dense understory of herbs, bamboo, shrubs, and tree saplings, in combination with a deep leaf-litter, provide habitat for endemic mammals of temperate forests such as the kodkod cat (*Leopardus guigna*), the austral opossum (*Dromiciops gliroides*), and the southern pudu (*Pudu puda*) [25–27]. Coarse woody debris is a critical habitat for great crested flycatchers (*Myiarchus crinitus*), amphibians, and reptile species in Loblolly Pine Forests [22,28] and moist forests in the Pacific Northwest [29]. For its part, Siitonen [30] and Grove [31] found a strong correlation between coarse woody debris and invertebrate species richness in boreal forests of Finland and lowland rainforests in Australia, respectively. Many invertebrates, cavity nesting birds, mammals, and herpetofauna depend largely on the availability of snags for their survival [24,29,30,32]. For example, Lohr [28] reported that the reduction of snags had detrimental effects on bird communities in Loblolly Pine forests. For its part, Ross et al. [33] described that the relative abundance and species richness of salamanders were correlated with the retention of tree basal area across forest stands in Pennsylvania. Other studies have reported a positive correlation between litter depth and richness and density of herpetofauna in tropical forests of Costa Rica [34]. Similarly, Uetz [35] reported that more species of spiders were found in areas with greater litter depth in North American temperate deciduous forests.

South American temperate forests, found between 35° - 55° south latitude, are considered one of the 35 Global Biodiversity Hotspots [36,37]. Despite their ecological importance, temperate forests of South America have experienced a long history of anthropogenic disturbances (deforestation and degradation) [37,38]. Nearly 70% of Chilean temperate forests have been lost due to large-scale and small-scale disturbances [39]. Management practices that enhance structural complexity should be a priority in South American temperate forest stands, mainly because a large proportion of their biodiversity is located in private lands beyond protected areas [40]. An index of stand structural complexity, that could guide forest management and conservation policy, has never been proposed for South American temperate forests.

In this paper, we develop an index of stand structural complexity and identify if anthropogenic disturbances (fire, logging, and livestock), and their combined presence, influence stand structural complexity in South American temperate forests. We specifically address the following questions: (1) Is it feasible to generate a simple but integrative index of stand structural complexity based on important attributes for the habitat of forest biodiversity? (2) Is there an effect of anthropogenic disturbances on stand structural complexity? (3) Which of the three tested anthropogenic disturbances, or their combined presence, affects stand structural complexity more strongly? Answering these questions will provide valuable theoretical insights into the assessment of structural complexity and forces disturbing forest stands in a Global Biodiversity Hotspot. Further, it will contribute with practical information for the design of policy decisions regarding forest management to enhance faunal biodiversity in areas under forest management beyond protected areas.

## Materials and Methods

### Study area

We conducted this study between 2011 and 2013 within the Araucarias Biosphere Reserve [41]. Specifically, we conducted vegetation surveys in an area of 2,585 km^2^ within the Villarica watershed in the Andean zone of the La Araucanía Region, southern Chile (39° 15’S 71°W). The area has a temperate climate with a short dry season (< 4 months) in the summer and mean precipitations of 1,945 mm/year. The vegetation comprises three vegetation types distributed along an elevational gradient from 200 masl (meters above sea level), up to the tree line at ~1,500 masl. At lower elevations (200–500 masl), forests are dominated by species like *Lophozonia obliqua* and *Nothofagus dombeyi*, associated mainly with *Laurelia sempervirens*, *Eucryphia cordifolia*, *Persea lingue*, and *Aextoxicon punctatum*. Mid-elevation forests (500–900 masl) are mixed stands dominated by the evergreen species *Saxegothaea conspicua*, *Laureliopsis philippiana*, and *N*. *dombeyi*. At higher elevations (> 900 masl), forests are dominated by *Araucaria araucana* and *Nothofagus pumilio* [42]. The majority of public protected areas and large forest tracks are at high elevations (> 700 masl) with a topography characterized by mountains and volcanoes. Valley floors are mostly used for agriculture in combination with human settlements in small to medium-sized villages and towns. This has caused lowland forest remnants (< 700 masl) to experience intense anthropogenic disturbances, mainly fires, logging, and livestock farming [43].

### Selection of structural attributes to be included in the index

**Fig 1:**
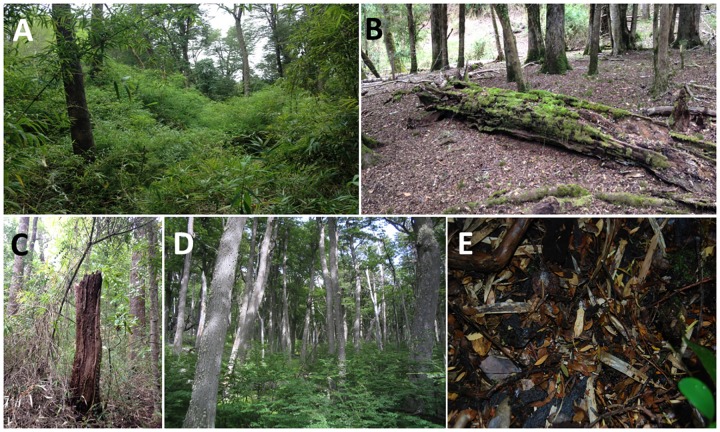
Five selected stand structural attributes included in this study: understory density (A), volume of coarse woody debris (B), snag density (C), stand basal area (D), and litter depth (E).

We constructed the index of stand structural complexity adapting the methodology proposed by McElhinny et al. [44]. Constructing an index of stand structural complexity requires a four-step approach involving: (1) establishing a comprehensive suite of stand structural attributes, (2) identifying the core set of attributes to be included in the index based on the specific purpose of the index, (3) surveying these attributes in a set of different stands, including different vegetation communities and habitat types, and (4) combining the core set of attributes into an additive index. To address point one, we selected those attributes that were considered a structurally important attribute for the habitat of mammals, birds, reptiles, amphibians, and invertebrates in both Andean temperate and forests elsewhere (Table 1). The core set of attributes should (i) have a low kurtosis (< 2), (ii) work as a surrogate for other structural attributes, and (iii) be easily measured in the field [44]. The five selected attributes included density of the understory, volume of coarse woody debris (CWD), density of dead standing trees (i.e. snags), stand basal area, and litter depth (Fig 1). These attributes can be readily surveyed in areas under forest management practices [10,44–46].

**Table 1:** List of five core attributes selected to be included in the index of stand structural complexity and their ecological importance for different faunal groups.

Structural complexity attribute	Description	References for South American temperate forests	References for forest ecosystems from elsewhere
Understory	A dense understory provides microclimatic conditions and food supply essential to understory birds. Some mammals require a dense understory for cover while stalking prey and breeding. The shade provided by a dense understory provides critical microhabitat resources for reptiles and moisture and temperature for amphibians. For invertebrates and several birds, a dense understory provides an important source of refuge to hide from predators.	[25,26,47–49]	[21,29,50–53]
Coarse woody debris	Coarse woody debris provides a favorable breeding habitat for several bird species. Small mammals can use CWD for travelling, foraging, and nesting. Many invertebrate species, especially saproxylic insects, depend on CWD for feeding. The presence of CWD plays an essential role as refuge for amphibian and reptile species.	[23,27,54–56]	[22,28,30,57,58]
Standing dead trees (i.e. snags)	Standing dead trees provide habitat for a range of forest-dwelling animal species. Birds and mammals use snags as shelter and breeding sites. Snags provide shade and litter inputs for amphibians as well as basking areas for reptiles. Snags provide high quality habitat and food resources for dead-wood dependent invertebrates.	[23,45,59–61]	[62–65]
Stand basal area	A relatively large stand basal area provides high availability of food resources to frugivorous and folivorous mammals and birds. A stand with a large basal area will provide a wide range of suitable microhabitats (e.g. tree cavities) for a series of invertebrate, amphibian, reptile and bird species.	[49,60,66,67]	[31,33,68]
Litter	Litter provides refuge for litter-dwelling invertebrates that are consumed by leaf-litter birds and mammals. This ground level attribute provides moist conditions that are essential for some forest small mammals. A relatively deep and dense leaf litter provides habitat niches required by reptile and amphibian species.	[69–73]	[34,35,74,75]

### Study design and forest sampling

We selected101 sites (N = 17 inside a public or private protected area) along an elevational gradient from 221 masl to 1,361 masl, including different habitat types such as old-growth forests (> 200 years old), secondary forests, arboreal shrublands, openfields, and exotic plantations. We selected the sites using ArcGIS 10.1 by identifying all the headwaters of smaller basins, within the Villarrica watershed, that could be accessed either by rural roads or hiking trails [76]. We randomly selected 13 out of the total 19 basins, placing the first site near the headwater of each basin [76]. We systematically located all the remaining sites every 1.5 km descending from the headwaters [76]. At each of the 101 sites, we established five vegetation plots (22.4 m diameter; 0.04 ha; N = 505 plots) using an L-shaped transect [76]. The first plot for each site was located at the vertex of the L-shaped transect. The remaining four plots were established with a distance of 125 m between each, along two 250 m lines directed outwards from the vertex [77]. Corporación Nacional Forestal (CONAF) allowed us to work on public protected areas (Permit # 11/2012 IX). In private areas, owners of the land gave permission to conduct the study on their properties. Field studies did not involve disturbing endangered or protected species.

In each vegetation plot, we measured a set of structural attributes, including: (1) understory density, (2) CWD, (3) number of snags, (4) tree diameter at breast height (DBH), and (5) litter depth (S1 File). We established two 11.2 m linear transects north to south from the center of the plot. Along this transect, we quantified understory density and litter depth at five points. We measured understory density by the number of contacts by the vegetation intercepting a 3 m vertical pole for each vertical meter up to 3 m height [78]. For each CWD with a diameter ≥ 7.5 cm, we measured the diameter at the center and the length. We calculated the volume of CWD (m^3^/ha) using the formula for volume of a cylinder. We measured tree DBH for all trees with DBH ≥ 12.5 cm. We counted all snags with DBH ≥ 12.5 cm and height ≥ 1.3 m [76]. We averaged the structural attributes within each plot in order to emphasize the unique structural conditions of each plot that differentiate it from adjacent areas [79]. In addition, we determined the presence or absence of anthropogenic disturbances (fire, logging, and livestock) for each plot. A series of high-severity human-set fires, that destroyed a large amount of stands in South American temperate forests, occurred in the early 20th century with the last stand-destroying event occurring between 1944 and 1945 [43,80,81]. Hence, fire occurring more than 70 years ago, was considered as the oldest disturbance and it was measured every time there were signs of fire-scars in a plot. We recorded logging when a plot had rests of logged wood or stumps without resprouts. We considered livestock activity when any signs of manure, pats, or browsed vegetation were present. We assigned each plot, in a 50 m radius, to one the following habitat types: (1) Old-growth forest (> 200 years old, N = 64), (2) mid-successional forest (< 70 years old with bamboo understory, N = 102), (3) mid-successional forest (< 70 years old with understory different than bamboo, N = 99), (4) mid-successional forest (< 70 years old without understory, N = 44), (5) early successional forest (< 20 years old, N = 75), (6) mixed shrubland (N = 87), (7) openfield (N = 28), and (8) exotic plantation (N = 6).

### Data analysis

#### Developing an index of stand structural complexity

To improve the distribution of the attributes showing a high kurtosis (> 2), we transformed the raw data to logarithm (x+1). We performed a regression analysis through quartiles to rescale each of the five selected stand structural attributes to a score ranging from 0 to 10. We set a score of 2.5, 5, 7.5, and 10 to the quartile midpoints corresponding to the 12.5, 37.5, 62.5, and 87.5 percentiles of the raw data distribution [10,44]. We attributed a maximum score of 10 to the 87.5 percentile while the equation was constrained so that the minimum score was 0. We obtained the index of stand structural complexity for each plot by adding all the rescaled values for the five measured structural attributes, with 0 being the minimum and 50 the maximum additive value. Thus, the total value of a plot with high structural complexity would approach a value of 50. Finally, we converted the index of stand structural complexity to percentage [10,44].

#### Relationship between disturbances and the index of stand structural complexity

Exotic plantations covered < 2% of the study area; therefore, we excluded exotic plantations from all analyses. We allocated each of the remaining plots (N = 499) to one of eight treatments representing anthropogenic disturbance, as follows: no disturbance or control (Treatment one, T1), fire (T2), logging (T3), livestock (T4), combined presence of fire and logging (T5), combined presence of fire and livestock (T6), combined presence of logging and livestock (T7), combined presence of fire, logging, and livestock (T8). We used non-parametric Kruskal-Wallis tests, with a Holm-Sidak multiple comparison post hoc approach, to assess significant differences in the mean values of the index of stand structural complexity among both the seven habitat types and the eight different disturbance treatments. We performed all the statistical analyses using the statistical software R [82].

## Results

### Stand structural attributes and stand structural complexity across habitat types

The mean values of the five selected stand structural attributes—understory density, volume of CWD, snag density, stand basal area, and litter depth—varied according to habitat type (Table 2). The highest mean values of understory density (7.55 ± 9.1 # contacts), volume of CWD (13.74 ± 23.79 m^3^/ha), stand basal area (75.28 ± 35.62 m^2^/ha), and litter depth (6.57 ± 3.7 cm) were measured in ‘old-growth forest’ plots while the highest mean value of snag density (46.02 ± 33.2 stems/ha) was measured in ‘mid-successional forest with bamboo understory’ plots. Conversely, the lowest mean values for each of the five stand structural attributes were measured in ‘openfield’ plots.

**Table 2:** Results showing the mean values (SD) of the five stand structural attributes included in the index of stand structural complexity and the values of index of stand structural complexity expressed in percentage in relation to the seven different habitat types.

Habitat type	Index of stand structural complexity (%)	Understory density (# contacts)	Volume of coarse woody debris (m^3^/ha)	Snag density (stems/ha)	Stand basal area (m^2^/ha)	Litter depth (cm)
Old-growth forest	80.03 (9.10)	7.55 (3.87)	13.74 (23.79)	46.02 (33.20)	75.28 (35.62)	6.57 (3.7)
Mid-successional forest (bamboo understory)	73.18 (11.82)	5.49 (2.13)	11.34 (16.88)	55.01 (75.62)	41.67 (33.07)	6.31 (3.36)
Mid-successional forest (other understory)	65.18 (10.32)	4.72 (2.61)	4.41 (8.68)	44.11 (68.58)	34.76 (19.24)	5.23 (2.63)
Mid-successional forest (no understory)	55.66 (13.75)	1.70 (1.24)	5.85 (15.01)	35.20 (53.15)	35.22 (17.95)	2.98 (2.43)
Early successional forest	54.21 (11.04)	3.58 (2.87)	2.30 (6.74)	17.26 (32.10)	16.71 (13.11)	4.30 (2.33)
Mixed shrubland	43.91 (11.24)	2.39 (2.13)	3.11 (20.32)	12.55 (38.11)	8.71 (13.91)	1.65 (1.83)
Openfield	34.38 (3.27)	0.42 (0.41)	1.18 (1.23)	0 (0)	2.21 (2.27)	0.41 (0.41)

**Fig 2:**
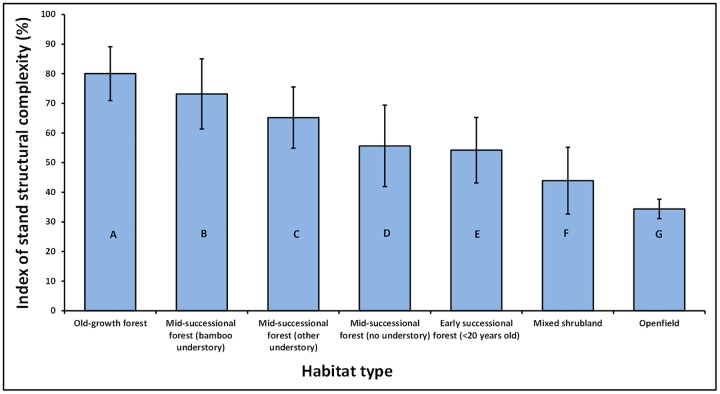
Relationship between the mean values of the index of stand structural complexity and seven habitat types. Bars with different letters were significantly different according to a Holm-Sidak post hoc test. Small bars are ± standard deviation.

There were significant differences in the mean values of the index of stand structural complexity among the seven habitat types (X^2^ = 302.0071, df = 6, p-value = 2.2e-16; Fig 2). The highest mean values for the index of stand structural complexity (80.03 ± 9.10) were measured in ‘old-growth forest’ while the lowest mean values for the index of stand structural complexity (34.38 ± 3.27) were measured in ‘openfield’ plots (Table 2).

For easier implementation in forest conservation and management programs, we calculated the values of the five stand structural attributes associated with predicted low (0–33%), moderate (34–66%), and high (67–100%) values of the index of stand structural complexity in Andean temperate forests (Table 3).

**Table 3:** Mean and range (in brackets) values of the five stand structural attributes associated with the estimated index of stand structural complexity categorized as low (0–33%), moderate (34–66%), and high (67–100%) in Andean temperate forests.

Stand structural attributes	Index of stand structural complexity		
	0–33%	34–66%	67–100%
Understory density (# contacts)	0.40 (0–1.0)	2.68 (1.20–4.40)	7.24 (4.6–20.2)
Volume of coarse woody debris (m^3^/ha)	0 (0–0)	0.85 (0–5.36)	22.24 (5.43–188.4)
Snag density (stems/ha)	0 (0–0)	7.71 (0–25.39)	94.44 (50.78–482.38)
Stand basal area (m^2^/ha)	1.11 (0–4.73)	18.48 (4.79–33.31)	61.22 (33.41–310.49)
Litter depth (cm)	0.39 (0–1.24)	3.03 (1.3–4.6)	7.53 (4.62–22)

### Anthropogenic disturbances and stand structural complexity

Ninety-five percent of the plots showed signs of anthropogenic disturbances (N = 475), with the combined presence of fire, logging, and livestock being the most common disturbance (N = 222; 44% of plots), and the combined presence of fire and livestock being the least common disturbance (N = 20). Only 24 plots did not show any signs of anthropogenic disturbance, from which 92% of these plots (N = 22) corresponded to ‘old-growth forests’.

The resulting mean values of the stand structural attributes that were selected to be included in the index of stand structural complexity varied according to the disturbance treatment (Table 4). The highest mean values of understory density (6.18 ± 2.87), volume of CWD (15.75 ± 30.35), and stand basal area (68.15 ± 28.34) were measured in plots that did not show any signs of disturbance. For its part, the highest mean values of snag density (60.93 ± 61.88) and litter depth (7.04 ± 3.1) were measured in plots that were subject to the combined presence of fire and livestock and fire respectively. Conversely, plots that were subject to the combined presence of logging and livestock showed the lowest mean values of understory density (2.72 ± 2.58) and volume of CWD (1.23 ± 2.24). Plots that were subject to the combined presence of fire, logging, and livestock showed the lowest mean values for litter depth (2.89 ± 2.76) and stand basal area (20.23 ± 24.46). The lowest mean value for snag density (18.13 ± 33.24) was measured in plots that were subject to livestock alone (Table 4).

**Table 4:** Results showing the mean (SD) values of both the stand structural attributes and the index of stand structural complexity for the eight disturbance treatments.

Treatment	Index of structural complexity (%)	Understory density (# contacts)	Volume of coarse woody debris (m^3^/ha)	Snag density (stems/ha)	Stand basal area (m^2^/ha)	Litter depth (cm)
No disturbance (control)	77.67 (11.15)	6.18 (2.87)	15.75 (30.35)	40.20 (35.84)	68.15 (28.34)	6.27 (3.49)
Fire	72.51 (13.44)	5.89 (2.84)	2.96 (4.40)	48.57 (42.56)	45.33 (31.78)	7.04 (3.10)
Logging	62.70 (13.72)	5.11 (3.53)	2.87 (8.72)	41.82 (67.28)	27.88 (18.60)	5.50 (2.85)
Livestock	54.74 (18.79)	4.01 (4.03)	3.38 (9.84)	18.13 (33.24)	32.36 (49.08)	3.57 (1.97)
Fire + Logging	71.63 (11.82)	5.60 (2.95)	7.84 (12.66)	50.78 (73.52)	44.31 (34.99)	6.31 (3.26)
Fire + Livestock	70.42 (12.16)	4.98 (2.39)	5.23 (7.32)	60.93 (61.88)	48.05 (19.81)	4.53 (1.69)
Logging + Livestock	53.48 (12.94)	2.72 (2.58)	1.23 (2.24)	23.94 (52.95)	30.18 (20.79)	3.58 (3.70)
Fire + Logging + Livestock	52.47 (16.28)	2.99 (2.81)	6.77 (19.04)	21.73 (44.32)	20.23 (24.46)	2.89 (2.76)

**Fig 3:**
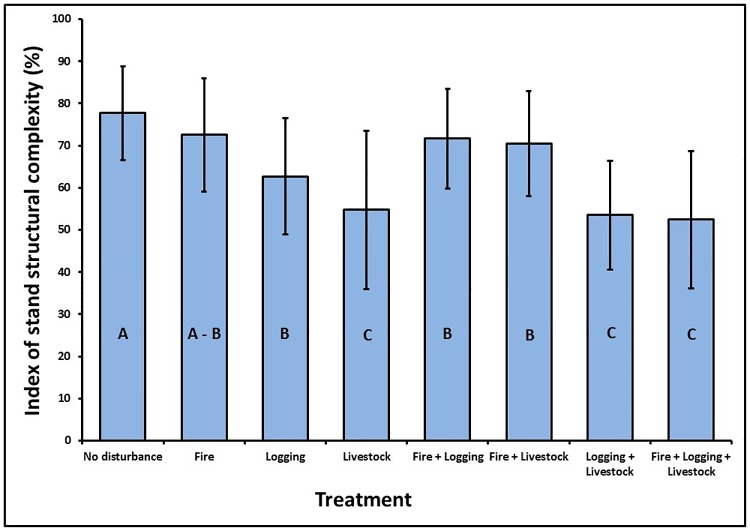
Relationship between the mean values of the index of stand structural complexity along a disturbance gradient. Bars with different letters were significantly different according to a multiple comparison post hoc test. Small bars are ± standard deviation.

The mean values for the index of stand structural complexity were significantly different among the eight disturbance treatments (X^2^ = 148.8512, df = 7, p-value = 2.2e-16) (Fig 3). The highest mean value of the index of stand structural complexity (77.67 ± 11.15) was measured in plots that did not show any signs of anthropogenic disturbances (control plots) while the lowest mean value of the index of stand structural complexity (52.47 ± 16.28) was measured in plots that were subject to the combined presence of fire, logging, and livestock. However, there were no significant differences in the mean values of the index of stand structural complexity among undisturbed plots (control) and plots that with the presence of fire alone (p = 0.73; Fig 3). The mean values of the index of stand structural complexity were lower in all the other six disturbance treatments compared to undisturbed plots. In addition, the presence of logging alone, livestock alone, the combined presence of logging and livestock, and plots with the presence of fire, logging, and livestock showed a significant difference with undisturbed plots (p < 0.05).

## Discussion

Indexes of stand structural complexity based on important attributes for the habitat of faunal species can be used to rank stands in terms of their potential contribution to biodiversity [4,44]. Anthropogenic disturbances, however, can degrade the composition and availability of these important structural attributes [16–18]. This work provides a novel index of stand structural complexity for South American temperate forests based on the quantification of key structural habitat attributes for faunal biodiversity: understory density, volume of CWD, snag density, stand basal area, and litter depth. Our results indicate that stand structural complexity is affected by logging and livestock, and the combined presence of fire, logging, and livestock. Nevertheless, the sole presence of fire occurring more than 70 years ago was the anthropogenic disturbance that showed the lowest effect on structural complexity. We suggest that specific forest management practices regarding the maintenance of structural attributes should be considered to enhance stand structural complexity in order to better conserve South American temperate forest biodiversity.

### Variation of attributes across habitat types helps determining stand structural complexity

To conserve temperate forest biodiversity, the maintenance of different habitat types must become a priority for forest managers [8]. However, early successional forest stages, shrublands, and open areas frequently dominate landscapes undergoing anthropogenic disturbances [7,15,83]. Old-growth stands tend to present a larger availability of structural attributes such as understory density, volume of coarse woody debris, snag density, stand basal area, and litter depth than other habitat types [4,7,8,84]. However, we found that some dead-tree attributes (snags) may be higher in successional forest stands [4,8].

Similar to McElhinny et al. [44], we suggest that there is no single ideal stand structure but, instead, a combination of different attributes could produce a variety of structurally complex stands. Díaz et al. [45] and Hansen et al. [4] suggested that understory density was positively associated with old-growth stands in South American and North American temperate forests, respectively. The values of understory density reported in this study may be of great conservation importance for some endemic species such as the threatened rufous-legged owl (*Strix rufipes*) and some understory specialist birds from the Rhynocriptidae and Furnariidae families, with densities positively associated with a dense understory vegetation [23,78,85].

Coarse woody debris (CWD) increases structural complexity [2,86], having a positive effect on forest biodiversity [55,56,87]. Our results concurs with previous studies reporting that the volume of CWD tends to accumulate in less disturbed habitats [8,19,87,88]. The retention of CWD after disturbances is critical for the conservation of different forests species. For example, Siitonen [30] proposed that reduction of CWD at a landscape level might led to the disappearance of more than 50% of saproxylic species in managed forests in Finland.

Regarding snag density, our results were similar to the ones reported by Franklin [8], Carmona et al. [89], and Veblen et al. [90], for northern and southern temperate forests. Even though disturbances kill many trees, they often do not consume all wood structure, and thus a large proportion of the dead remnants (e.g. snags) are available for faunal species after disturbance. We found the highest mean values of snag density in ‘mid-successional forest with bamboo understory’. These snags readily become habitat legacies of disturbance events, and generally positively influence breeding site selection and fecundity of several faunal species [9,49,91].

Armesto and Figueroa [92] showed that stand basal area was directly correlated with less disturbed habitats. These findings support our results because the higher values of stand basal area were measured in old-growth plots. In addition, our values of stand basal area for Andean temperate forests are similar to the ones reported for other areas in southern temperate forests, where stand basal area was considerably higher in old-growth stands in comparison to early and mid-successional stands [45,89,93]. Similarly, basal area in northern temperate forests was an important structural attribute to discriminate among habitat types [88].

The high mean values of litter depth measured in this study for old-growth and mid-successional forest with bamboo understory plots illustrate the importance of bamboo plants for increasing stand structural complexity. Armesto and Fuentes [94], Christie and Armesto [87], and Veblen et al. [95] also observed changes in stand structure with higher litter depth produced by bamboo leaves. Spies and Franklin [88] reported higher values of litter depth in old-growth stands in comparison to young stands for northern temperate forests. However, even though litter depth is an easily measurable attribute, few studies have been undertaken in South American temperate forests regarding litter. This makes difficult to make comparisons among our results and other studies.

For its part, the lowest mean values of the five stand structural attributes measured in openfield plots are a direct consequence of land clearance for agriculture and livestock farming [80,92,96]. A common practice of land clearing is to remove CWD and snags for usage as timber-derived products or firewood [8,89].

### Anthropogenic disturbances and stand structural complexity in Andean temperate forests

Stand structure has been shaped by a continuous history of anthropogenic disturbances in South American temperate forests [80,96]. The large extent of these disturbances helps to explain the high proportion of plots (N = 475; 95% of plots) that were subject to disturbance [43]. When comparing the writings of Charles Darwin about South American temperate forests with forest condition at present (150 years after Darwin′s observations), Willson and Armesto [97] stated that forest structural complexity described by Darwin is greatly absent nowadays because of logging, burning, and land clearing for agriculture.

Different authors have acknowledged the resilience capacity of temperate forests to moderate levels of anthropogenic disturbances [8,37,98]. Our results show that plots burned more than seven decades ago can achieve high to moderate levels of structural complexity, suggesting that South American temperate forests may have some resiliency to fire. Similar results were found for the structure of abandoned pastures in Puerto Rico, where basal area, aboveground biomass, and species richness in secondary forest achieved similar values to those in old-growth forest 40 years after fire [99]. A greater array of structural attributes would provide greater ecosystem resilience [100] and recovery after anthropogenic disturbances [101]. This hypothesis is supported by previous studies where the values of understory density and basal area increased in relation to the elapsed time since disturbance [45]. In addition, our unexpected high mean values of post-fire understory density may be explained by the proliferation of a dense bamboo understory. Following fire events, bamboos have been described to dominate forest understory, forming extensive thickets impeding the regeneration of other vegetative species [80,102].

The continuous cutting of snags and removal of logs for firewood, may be a potential explanation for the lower values of the index of stand structural complexity in plots subject to the combined presence of fire and logging. For instance, the low values of the volume of CWD measured in this study for plots that were subject to fire may be explained by the removal of logs and snags by landowners to be used or sold as firewood; a very common practice in southern Chile [89,98,103]. In addition, logging practices have resulted in the alteration and elimination of the understory due to the opening of roads for timber extraction with animals and machinery [104]. Studies in northern temperate forests have demonstrated that snags were three to five times more dense in undisturbed plots in comparison with logged plots [4]. Similarly, Aravena et al. [98] indicated that basal area increased significantly in undisturbed plots in comparison to plots that showed the combined presence of fire and logging. This supports findings of a reduction in stand structural attributes such as understory density, volume of CWD, and snag density in plots that were subject in areas under simultaneous fire and logging activities [4,50,89,105,106].

Interestingly, there was not a significant difference in the mean values of the index of stand structural complexity between plots that were subject to the presence of logging alone or in combination with fire. This result is comparable to previous studies [89,107–109], in which mean values of CWD volume, snag density, and basal area showed little differences between plots showing signs of fire in comparison to plots showing the combined presence of fire and logging. However, when assessing the difference between the mean values of structural attributes reported in those studies with our findings, the results should be viewed with caution because the elapsed time since the disturbance may have been different. For instance, the post-fire plots measured in this study may have been immediately abandoned after the occurrence of the last catastrophic fire that occurred 70 years ago, having a longer recovery time since disturbance.

The introduction of livestock by European colonists has had a detrimental effect on stand structure by reducing stand attributes such as understory density, basal area, and litter depth [20,43,81,90]. Our results correspond with this statement as they show that the presence of livestock—solely or in combination with other disturbances—is the most important factor decreasing stand structural complexity in South American temperate forests. Tasker and Bradstock [110] reached the same conclusion when studying the influence of livestock grazing on forest understory in New South Wales, Australia. Similarly, livestock had a stronger negative effect on forest regeneration in comparison to logging in the composition and structure of southern temperate forests [111].

Grazing activity by livestock not only affects understory density but other structural attributes. For example, low values for stand basal area, and for size and abundance of woody plants have been reported for forests with a high density of livestock [93,112]. Also, regeneration of trees (e.g. *A*. *araucana*) is significantly affected by the presence of livestock [20]. Livestock also affects understory structure by grazing and trampling the herbaceous layer [113]. For example, Rummell [114] showed that grazing by livestock reduced understory vegetation by 45–61% in ponderosa pine forests. In Argentina, understory density—mainly of bamboo—was significantly reduced in highly grazed stands in comparison to less disturbed stands [112].

We did not find information on the effects that livestock has on CWD volume or snag density in South American temperate forests. However, our results suggest that the reduction of understory density and basal area by livestock is indirectly affecting other structural attributes such as the volume of CWD, snag density, and litter depth. For instance, the lowest mean values of litter depth reported in this study were measured in plots that were subject to the presence of livestock. This result may be associated with the fact that livestock grazing of plant biomass located above ground minimizes the quantity of biomass for litter conversion [113]. Similarly, Hayes and Holl [115] reported that litter depth was significantly lower in grazed sites in comparison to ungrazed sites when investigating the impact of cattle grazing on a coastal prairie plant community in California. On the other hand, the low mean values of volume of CWD and stand basal area in plots that were subject to livestock may be due to the practice of removing bamboo understory as well as other woody debris for aesthetic and logging activity reasons [48].

### Recommendations for management

In South American temperate forests, rapid degradation at the forest stand level and the lack of protection beyond protected areas are calling for novel ways of managing forests to prevent the loss of biodiversity. Our results suggest that temperate forest biodiversity will be benefited if management initiatives, promoting the retention of structural attributes and the reduction of anthropogenic disturbances, are implemented.

We show that there is no single ideal stand structure or habitat type that maximizes structural complexity. However, our results indicate that forest management schemes should aim to retain structural attributes including understory density (7.2 ± 2.5 # contacts), volume of CWD (22.4 ± 25.8 m^3^/ha), snag density (94.4 ± 71.0 stems/ha), stand basal area (61.2 ± 31.4 m^2^/ha), and litter depth (7.5 ± 2.7 cm). These desired values of structural attributes might be reached by retaining large trees (DBH > 53 cm) and retaining a dense bamboo understory. The application of the index of stand structural complexity in order to guide conservation management will likely benefit temperate forest biodiversity [45,48,85].

## Supporting Information

S1 File Vegetation plot data containing plot numbers, habitat type, presence of fire, logging, and livestock as well as the following stand structural attributes: understory density (# contacts), volume of coarse woody debris (m^3^/ha), stand basal area (m^2^/ha), litter depth (cm), snag density (stems/ha), and the values for the index of stand structural complexity (%) for each plot. (XLSX)
