# Correction: Influence of Anthropogenic Disturbances on Stand Structural Complexity in Andean Temperate Forests: Implications for Managing Key Habitat for Biodiversity

**DOI:** 10.1371/journal.pone.0174147

**Published:** 2017-03-13

**Authors:** The PLOS ONE Staff

The following is missing from the Funding section: This study was supported by Comisión Nacional de Investigación Científica y Tecnológica grant CONICYT/FONDECYT de Iniciación 11160932 to JTI. The publisher apologizes for the mistake.
